# Shaoyao-Gancao Decoction Promoted Microglia M2 Polarization via the IL-13-Mediated JAK2/STAT6 Pathway to Alleviate Cerebral Ischemia-Reperfusion Injury

**DOI:** 10.1155/2022/1707122

**Published:** 2022-06-17

**Authors:** Juanjuan Lu, Jie Wang, Long Yu, Rong Cui, Ying Zhang, Hanqing Ding, Guofeng Yan

**Affiliations:** ^1^Department of Rehabilitation, Shanghai Xuhui Central Hospital, Shanghai 200031, China; ^2^School of Kinesiology, Shanghai University of Sport, Shanghai 200438, China; ^3^Academy of Integrative Medicine, Shanghai University of Traditional Chinese Medicine, Shanghai 201203, China; ^4^School of Medicine, Shanghai Jiao Tong University, Shanghai 200025, China

## Abstract

Microglia in the penumbra shifted from M2 to M1 phenotype between 3 and 5 days after cerebral ischemia-reperfusion, which promoted local inflammation and injury. Shaoyao-Gancao Decoction (SGD) has been found to result in a significant upregulation of IL-13 in the penumbra, which has been shown to induce polarization of M2 microglia. There was thus a hypothesis that SGD could exert an anti-inflammatory and neuroprotective effect by activating IL-13 to induce microglia polarization towards M2 phenotype, and the purpose of this study was to explore the influence of SGD on microglia phenotype switching and its possible mechanism. Rats who received middle cerebral artery occlusion surgery (MCAO) were treated with SGD for 3 or 6 days, to investigate the therapeutic effect and the underlying mechanism of SGD for cerebral ischemia-reperfusion injury (CI/RP). The results indicated that SGD improved neurobehavioral scores and reduced apoptosis. Furthermore, SGD significantly decreased M1 microglia and M1-like markers, but increased M2 microglia and M2 markers. Moreover, higher levels of IL-13 and ratios of p-JAK2/JAK2 and p-STAT6/STAT6 were found in the SGD group compared to the MCAO. In conclusion, it was verified that SGD prevented injury by driving microglia phenotypic switching from M1 to M2, probably via IL-13 and its downstream JAK2-STAT6 pathway. Given that no further validation tests were included in this study, it is necessary to conduct more experiments to confirm the reliability of the above results.

## 1. Introduction

Stroke is one of the most serious diseases worldwide, and ischemic stroke (IS) is the most common, leading to high disability and death rates and adding a serious social economic load. In clinical, the recombinant plasminogen activator is widely recognized as the most effective treatment for IS at present, but it has a narrow time window and could increase the risk of symptomatic intracerebral hemorrhage [1]. Other commonly used neuroprotective agents also have side effects. For instance, transient hypotension is most frequently observed in patients treated with human urinary kallidinogenase (HUK) [2]. Thus, it is necessary to explore the new and effective therapy for treating IS.

It is worth noting that reperfusion injury after IS treatment is inevitable. More attention has been paid to the immunoinflammatory response during cerebral ischemia-reperfusion (CI/RP) injury, which is characterized by microglia activation and production of inflammatory cytokines and chemokines [3]. Microglia can be activated into M1 and M2 phenotypes, depending on the stimulus [4]. M1 microglia display an inhibitory influence on brain recovery by releasing various proinflammatory cytokines. On the contrary, M2 microglia contribute to a beneficial process, such as reduced inflammation, tissue repair, and cell debris removal [5, 6]. The phenotypic switch of microglia may be induced after injury caused by cerebral ischemia [7]. In the acute phase, M2 phenotype microglia were abundantly expressed in the core of the infarct, while substantial numbers of M1 microglia aggregated in the penumbra. Subsequently, M1 microglia increased at 3 days and peaked at 14 days after ischemia. A declining trend in M2 microglia began at 7 days and lasted until 14 days [8]. Therefore, promoting an M1 to M2 phenotypic shift in the penumbra during this period might be a potential therapeutic target to the alleviation of inflammatory injury and to exert neuroprotective effects against CI/RP injury.

Shaoyao-Gancao Decoction (SGD), a classic Chinese herbal formula, is derived from the Treatise on Febrile Diseases, written by Zhang Zhong Jing [9]. It has distinct advantages of producing spasmolysis, analgesia, and anti-inflammatory actions [10]. Recent clinical studies confirmed that SGD could improve motor ability, reduce spasm, pain, and swelling in patients with hemiplegia after stroke [11, 12]. However, the precise mechanism of the protective effect of SGD against CI/RP injury has not been unequivocally determined. For all we know, several studies have demonstrated that SGD and active components, including paeoniflorin, glycyrrhizin, and liquiritin, play neuroprotective roles by inhibiting inflammatory responses in neurological disorders [13, 14]. For example, SGD has been shown to be effective in learning and cognitive improvements by decreasing neuroinflammation in Alzheimer's disease [15]. The beneficial effects of paeoniflorin and glycyrrhizin on brain recovery were attributed to the reduction in expression of proinflammatory factors, such as nuclear factor-*κ*B (NF-*κ*B), interleukin- (IL-) 1*β*, and tumor necrosis factor- (TNF-) *α* [16, 17]. In summary, SGD and bioactive constituents provide protective effects by suppressing proinflammatory responses. Whether the SGD-induced positive influence on CI/RP recovery is associated with the anti-inflammatory feature of SGD still remains unknown and needs further studies.

In our pre-experiments, a significant upregulation of IL-13, an anti-inflammatory factor, in the penumbra was measured after 6-day gavage treatment with SGD. IL-13, also a stimulus that induces M2 microglia, binds to its receptor (IL-13 R*α*1) and activates the Janus kinase/signal transduction and activator-6 pathway (JAK/STAT6), polarizing microglia towards the M2a phenotype [18–20]. A previous study suggested that IL-13 administration could elevate the ratios of M2 microglia and improve the long-term sensorimotor deficit after IS [21]. Therefore, it is reasonable to presume that IL-13 might function in promoting microglia polarization to the M2 phenotype in CI/RP rats after SGD treatment.

The purpose of this study was to investigate the anti-inflammatory effect of SGD and its possible mechanism in CI/RP rats. Specifically, we evaluated the expression of M1 and M2 microglia in the penumbra after SGD treatment and explored whether the induction of M2 polarization by SGD was mediated via IL-13 and its downstream pathway JAK2/STAT6.

## 2. Materials and Methods

### 2.1. Animals

Specific-pathogen-free male Sprague Dawley rats (aged 8-10 weeks, 250-320 g) were purchased from Slack Experimental Animal Company (Shanghai, China). All animals were cared for in line with the guidelines legislated by the National Institutes of Health. The rats were divided into 3 groups, namely, SGD, MCAO, and sham groups in a random order. Permission for the experimental scheme from the Ethics Committee of Shanghai Jiao Tong University was granted (Ethics No. 2019027).

### 2.2. CI/RP Model

**Figure 1:**
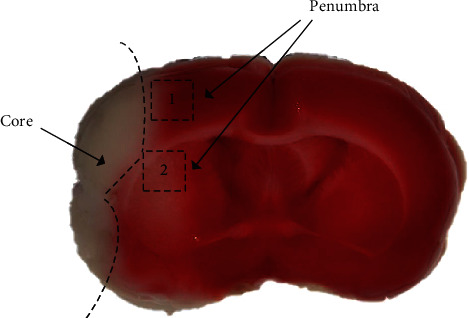
The penumbra and infarct core in rats after MCAO. The penumbra in the cortex was indicated with the black dashed box marked “1,” and the striatum was marked “2,” which were selected as the cortex and striatum for immunofluorescence staining and immunohistochemistry. Penumbra tissue was also used for qRT-PCR, Luminex multiplex assay, and western blotting.

Rats in the SGD and MCAO groups underwent middle cerebral artery occlusion- (MCAO-) reperfusion surgery according to the previously described method [22]. During surgery under deep general anesthesia, expose the right common carotid artery branch and then insert a filament with a silicone tip of 1.8-2.0 cm into the internal carotid artery (ICA) to stop blood flow. After 120 min, the filament was removed to allow reperfusion. The Zea-Longa test [22] was carried out at 24 h after the operation, and only rats with a score of 1-3 were accepted for later research. The same surgical procedures took place in rats in the sham group, except for the filament being kept in the ICA. The penumbra in the cortex and striatum for molecular experiments is shown in Figure 1.

### 2.3. Preparation of SGD

SGD was prepared from Paeoniae Radix alba slices (batch number: 2004086) and honey-fried Glycyrrhizae Radix et rhizoma pieces (batch number: 2005072) by the water decoction method, which were obtained from Sichuan Neautus Traditional Chinese Medicine Co., Ltd. (Sichuan, China). The crude drug concentration was 1.05 g/mL, which was stored at 4°C for future experiments.

### 2.4. High-Performance Liquid Chromatography (HPLC) Analysis

Seven active ingredients, including oxypaeoniflorin, paeoniflorin, albiflorin, liquiritin, glycyrrhizin, isoliquiritin, and liquiritigenin, were used for the quantity control of SGD and were relatively quantified by HPLC on an Agilent HPLC-DAD system. The optimum separation conditions were achieved by employing acetonitrile (A)-0.1% aqueous phosphoric acid (B) as the mobile phase, with the column temperature at 30°C. The injection amount was 10 *μ*L, and the flow velocity was 1 mL/min.

### 2.5. Experimental Design

Rats in the SGD group were treated with 4 mL of SGD by gavage once a day from 24 h after the surgery, lasting 3 days or 6 days, while rats in the other 2 groups were given saline solution in the same volume, frequency, and treatment times.

### 2.6. Neurological Scores

Neurobehavioral dysfunction of rats in the 3 groups was assessed by an operator who was blind to the group allocation using the modified neurological severity score (mNSS) [23] at 1 day, 4 days, and 7 days after MCAO. mNSS is an 18-point assessment, containing motor, sensory, reflex and balance examinations, and higher scores predict worse behavioral performance.

### 2.7. Quantitative Real-Time Polymerase Chain Reaction (qRT-PCR) Analysis

The procedure for extracting total RNA from the penumbra and the reverse transcription process followed the manufacturer's protocol. qRT-PCR for the content of IL-4, IL-13, IL-1a, IL-1b, IL-6, and IL-10 was performed using an Applied Biosystems system (7500 Real-Time PCR Software, Waltham, MA, USA). The kits involved were purchased from Tiangen Biotech (Beijing, China). U6 served as internal control and the forward primer sequences were listed in Table 1, which refers to the records of previous studies [24–28]. The 2^−*ΔΔ*CT^ method was employed to detect the target genes' mRNA expression [29].

### 2.8. Luminex Multiplex Assay

The Bio-Plex Pro™ Rat Cytokine 23-Plex Assay (12005641, Bio-Rad, Hercules, CA, USA) was adopted to detect the concentration of multiple cytokines (IL-4, IL-13, IL-1a, IL-1b, IL-6, and IL-10) in the penumbra on a Luminex 200 instrument (Thermo Fisher Scientific, Waltham, MA USA). The unit of protein expression in the study was pg/mg.

### 2.9. Western Blotting

The penumbra tissue was treated with RIPA lysis buffer (Yamei, Suzhou), and the protein concentration was measured. 10% sodium dodecyl sulfate-polyacrylamide gel electrophoresis (SDS-PAGE) was used to separate the target proteins, which were then transferred to a nitrocellulose membrane. The membrane was blocked with 5% BSA for 1 h and then incubated with 5% BSA containing corresponding primary antibodies, including JAK2 (YT2426, Immunoway, Plano, TX, USA) (1 : 400), p-JAK2 (3776, Cell Signaling Technology, Danvers, MA, USA) (1 : 500), STAT6 (YT4454, Immunoway) (1 : 400), p-STAT6 (YP0256, Immunoway) (1 : 400), Bcl-2 (YT0470, Immunoway) (1 : 500), Bax (YT0455, Immunoway) (1 : 500), and GAPDH (YM3029, Immunoway) (1 : 5,000) on a shaker at 4°C overnight. On the second day, Tris-buffered saline-tween (TBST) was used to rinse the membranes 3 times, which were then incubated with secondary antibodies (RS23710, RS23920, Immunoway) (1 : 10,000) for 1 h. The bands were observed using an Odyssey Infrared Imaging System 3.0.29 (LICOR, Nebraska, USA).

### 2.10. Double Immunofluorescence Staining

Brains sections were immersed in 0.1% Triton X-100 for 10 min, blocked for 1 h, and then exposed to primary antibodies at 4°C overnight, including rabbit anti-Iba1 (019-19741, Wako, Chuo-Ku, Osaka, Japan) (1 : 500) and mouse anti-CD68 (MCA341R, Bio-Rad) (1 : 50) or mouse anti-Arg-1 (sc-271430, Santa Cruz Biotechnology, Dallas, TX, USA) (1 : 25). Subsequently, the incubation solution was replaced by goat anti-rabbit IgG (A-21428, Thermo Fisher Scientific) (1 : 1000) and -mouse IgG (A-11029, Thermo Fisher Scientific) (1 : 500). Finally, sections were counterstained with DAPI (G1012, Servicebio, Wuhan). Images were recorded using a ZEN imaging system (Zeiss, Jena, Germany) at ×200 magnification.

### 2.11. Immunohistochemistry

After 3 rinses with PBS, sections were soaked in mixed solution containing PBS, methanol, and 30% H_2_O_2_ for 30 min. Caspase-3 (ab2302, Abcam, Cambridge, UK) (1 : 200) served as the primary antibody, followed by incubation with the appropriate secondary antibody (65-6140, Thermo Fisher Scientific) (1 : 1,000) for 2 h. When observed under a Zeiss confocal microscope (LSM 800) at ×200 magnification, the positive cells appeared brown. The number of caspase-3 positive cells was quantified using Image-J 1.51 software (Media Cybernetics Inc. Co.).

### 2.12. Statistical Analysis

Data processing was executed using SPSS ver. 26.0 (IBM SPSS Statistics, Chicago, IL, USA), and the corresponding charts were constructed using GraphPad Prism version 8.3.0 (GraphPad Prism, San Diego, CA, USA). The results are given as the mean ± standard deviation (SD). Student's *t*-test was applied to verify any differences between the 2 groups. One-way ANOVA was performed to determine the difference among 3 groups, and an LSD test for post hoc comparison was then conducted. A *P* value < 0.05 was considered a statistically significant difference.

## 3. Results and Discussion

### 3.1. HPLC Analysis of Seven Components in SGD

**Figure 2:**
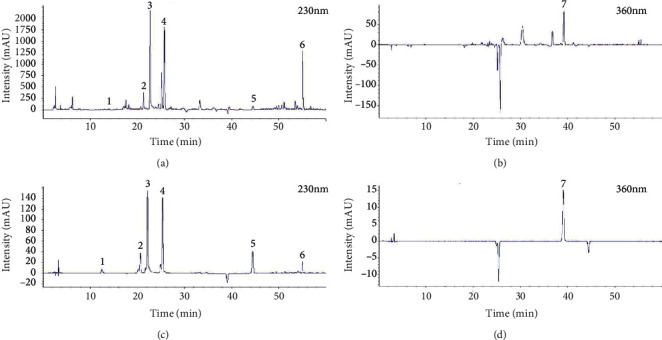
HPLC chromatograms of seven analytes in SGD and standard mixture. (a, b) HPLC chromatograms of seven analytes in SGD at 230 nm (a) and 360 nm (b). (c, d) HPLC chromatograms of seven analytes in a standard mixture at 230 nm (c) and 360 nm (d). The meaning of numbers was as follows: (1) oxypaeoniflorin, (2) albiflorin, (3) paeoniflorin, (4) liquiritin, (5) liquiritigenin, (6) glycyrrhizin, and (7) isoliquiritin.

The SGD chromatogram was established by HPLC, and the concentrations of 7 components were determined simultaneously. As shown in Figure 2, oxypaeoniflorin, albiflorin, paeoniflorin, liquiritin, liquiritigenin, and glycyrrhizin reached peaks at 230 nm, and the retention time is 13.923 min, 21.284 min, 22.661 min, 25.692 min, 44.482 min, and 55.069 min, respectively. Isoliquiritin peaked at 360 nm, and the corresponding retention time was 39.193 min. According to the linear regression equations of the 7 analytes (Table 2), the relative content of the seven active constituents of SGD is listed in Table 3.

### 3.2. SGD Alleviated Neurological Impairments following CI/RP in Rats

**Figure 3:**
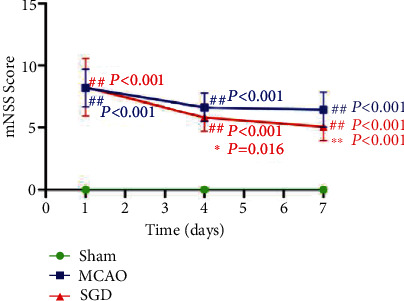
SGD alleviated neurological impairments following CI/RP in rats. Before humane sacrifice, all rats in the sham, MCAO, and SGD groups underwent the neurological evaluation named mNSS at 1 d, 4 d, and 7 d post-surgery. Data are expressed as mean ± SD, *n* = 16 rats per group. ^##^*P* < 0.01 vs. sham; ^∗^*P* < 0.05, ^∗∗^*P* < 0.01 vs. MCAO.

To investigate whether SGD had beneficial effects on neurological behavior after MCAO, rats were treated with SGD for 3 days or 6 days post-surgery. As shown in Figure 3, mNSS scores showed no significant differences on day 1 between the MCAO and SGD groups, while significant improvements in neurological deficits were observed after SGD treatment lasting 3 days (*P* < 0.05) and 6 days (*P* < 0.01). The score of rats in the sham group was 0 as they had no neurobehavioral deficits. These data suggested that SGD administration could lessen functional disturbances in an effective manner after CI/RP.

### 3.3. SGD Boosted Microglia Polarization from M1 to M2 after CI/RP Treatment of Rats

**Figure 4:**
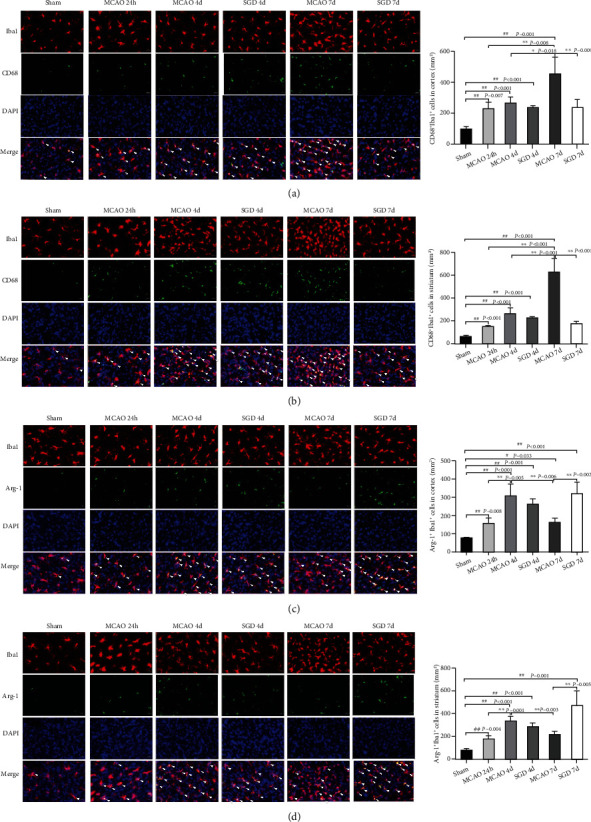
SGD boosted microglia polarization from M1 to M2 after CI/RP treatment of rats. Double immunostaining (×200) for Iba1 and CD68 or Arg-1 was performed in brain sections from the sham, MCAO, and SGD groups at three time points. The white arrows in the merged figures indicated double positive cells. (a, b) Representative images of Iba1 (red) and CD68 (green) labeling, followed by stained with DAPI (blue) in the cortex (a) and striatum (b), and statistical results were presented on the right side. (c, d) Representative images of Iba1 (red) and Arg-1 (green) labeling, followed by stained with DAPI (blue) in the cortex (c) and striatum (d), and quantitative analysis of double-positive cell counting were listed on the right side. Bar = 20 *μ*m for all images, *n* = 3 for each group. ^#^*P* < 0.05, ^##^*P* < 0.01 vs. sham; ^∗^*P* < 0.05, ^∗∗^*P* < 0.01 vs. MCAO.

To understand the changes in microglia phenotypes after 3-day and 6-day SGD treatment in MCAO rats, we performed double immunostaining of Iba1 with CD68 (M1 microglia) or Iba1 with Arg-1 (M2 microglia) in the cortex and striatum. As shown in Figure 4, in comparison with the sham group, M1 microglia after MCAO showed a growing trend from 24 h to 7 days after surgery both in the cortex (*P* < 0.01, Figure 4(a)) and in the striatum (*P* < 0.01, Figure 4(b)), while M2 microglia in the cortex and striatum remained increasing at 24 h (*P* < 0.01) and 4 days (*P* < 0.01), then decreased but were still higher at 7 days in the cortex (*P* < 0.05) in the MCAO group (Figures 4(c) and 4(d)). Furthermore, there were smaller numbers of CD68^+^ microglia in the cortex (*P* < 0.01) and striatum (*P* < 0.01) at 6 days in the SGD group compared to the MCAO group. In contrast, a significant increase in Arg-1 microglia was observed in the cortex (*P* < 0.01) and striatum (*P* < 0.01) at 7 days in the SGD group compared to the MCAO group. In general, SGD suppressed M1 microglia and boosted M2 microglia following CI/RP.

### 3.4. SGD Induced a Decline in M1-Like Markers and an Elevation of M2-Like Markers

**Figure 5:**
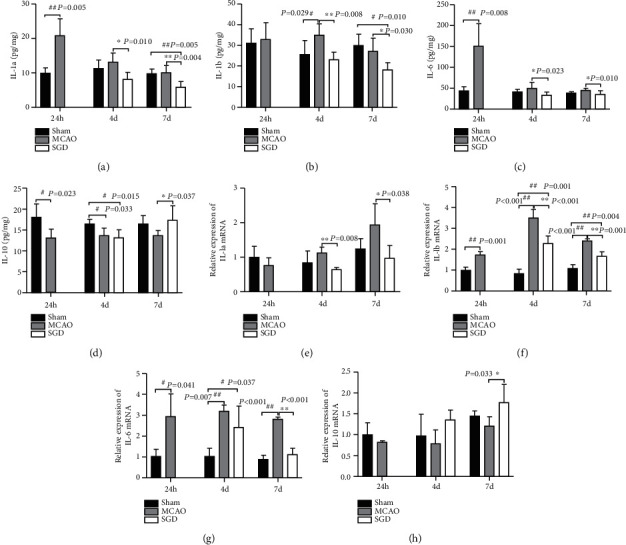
SGD induced a decline in M1-like markers and an elevation of M2-like markers. Luminex and qRT-PCR were performed to determine the expression of phenotypic markers in the sham, MCAO, and SGD groups at 24 h, 4 d, and 7 d after CI/RP. The corresponding statistical histograms of M1 markers, IL-1a (a, e), IL-1b (b, f), IL-6 (c, g), and the M2 marker IL-10 (d, h) are displayed. Results are presented as the mean ± SD, *n* = 4 − 5 for Luminex, and *n* = 3 − 4 for qRT-PCR in each group. ^#^*P* < 0.05, ^##^*P* < 0.01 vs. sham; ^∗^*P* < 0.05, ^∗∗^*P* < 0.01 vs. MCAO.

Subsequently, the expression of M1-like markers (IL-1a, IL-1b, and IL-6) as well as M2-like marker (IL-10) was tested by Luminex and qRT-PCR to give a further verification on phenotypic transformations caused by the SGD treatment. As shown in Figures 5(a) and 5(c) by Luminex, compared to the sham group, IL-1a and IL-6 both peaked at 24 h (*P* < 0.01) and then exhibited a downward trend after MCAO, while IL-1b reached its highest level 4 days after surgery (*P* < 0.05, Figure 5(b)). As an anti-inflammatory cytokine, IL-10 concentration decreased significantly at 24 h and 4 days after MCAO (*P* < 0.05, Figure 5(d)). The qRT-PCR results showed that the highest level of IL-1a appeared at 7 d (Figure 5(e)), and IL-1b and IL-6 seemed to reach their peaks at 4 d (*P* < 0.01, Figures 5(f) and 5(g)) post-surgery, while the mRNA level of IL-10 was still decreased in the MCAO group compared to the sham group (Figure 5(h)). These trends were reversed by SGD treatment. To be specific, significant reduction in the concentrations of IL-1a, IL-1b, and IL-6 was measured in the 3-day (IL-1a: *P* < 0.05 for Luminex and *P* < 0.01 for qRT-PCR; IL-1b: *P* < 0.01 for Luminex and qRT-PCR; IL-6: *P* < 0.05 for Luminex) and 6-day SGD groups (IL-1a: *P* < 0.01 for Luminex and *P* < 0.05 for qRT-PCR; IL-1b and IL-6: *P* < 0.05 for Luminex and *P* < 0.01 for qRT-PCR). On the contrary, there is significant increase in the level of IL-10 after 6-day SGD (*P* < 0.05 for Luminex and qRT-PCR). Combined with results from immunostaining, Luminex, and qRT-PCR, we drew the conclusion that SGD could drive microglia polarization from the M1 phenotype towards the M2 phenotype to exert protective effects against CI/RP injury.

### 3.5. SGD Increased the Expression of IL-13 following CI/RP in Rats

**Figure 6:**
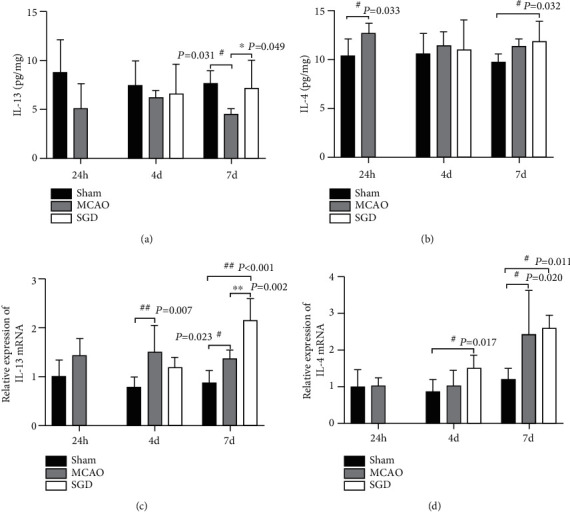
SGD increased the expression of IL-13 following CI/RP in rats. The levels of IL-13 and IL-4 in the penumbra were detected by Luminex and qRT-PCR on days 1, 4, and 7 post-surgery among the sham, MCAO, and SGD groups. (a, b) Comparative results of protein level of IL-13 (a) and IL-4 (b). (c, d) Statistical analysis of mRNA expression of IL-13 (c) and IL-4 (d). Results are presented as the mean ± SD, *n* = 4 − 5 for each group. ^#^*P* < 0.05, ^##^*P* < 0.01 vs. sham; ^∗^*P* < 0.05, ^∗∗^*P* < 0.01 vs. MCAO.

It is well established that M2 microglia can be induced by IL-4 and/or IL-13 stimulation. In the present study, we detected the expression of IL-4 and IL-13 in the sham, MCAO, and SGD groups 1, 4, and 7 days after surgery. Luminex results revealed that a significant increase in IL-13 expression occurred in the SGD group compared to the MCAO group on day 7 (*P* < 0.05, Figure 6(a)), while the difference in IL-4 expression was not notable at the indicated time points (*P* > 0.05, Figure 6(b)). These findings are consistent with the results from qRT-PCR that SGD treatment for 6 days markedly elevated the mRNA expression of IL-13 (*P* < 0.01, Figure 6(c)), rather than IL-4 (*P* > 0.05, Figure 6(d)). It is thus assumed that IL-13 participated in SGD-evoked M2 microglia polarization.

### 3.6. SGD Activated JAK2/STAT6 Pathway Involved in IL-13-Mediated Microglia Polarization following CI/RP in Rats

**Figure 7:**
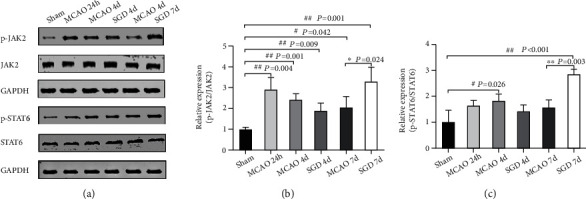
SGD-activated JAK2/STAT6 pathway is involved in IL-13-mediated microglia polarization following CI/RP in rats. The protein expression of JAK2, p-JAK2, STAT6, and p-STAT6 in extracts of the penumbra among the sham, MCAO, and SGD groups was analyzed by western blotting at the 3 indicated time points. (a) Representative bands of JAK2, p-JAK2, STAT6, p-STAT6, and GAPDH are shown. In this section, a statistical process was carried out on the ratios of p-JAK2/JAK2 (b) and p-STAT6/STAT6 (c). The results are presented as mean ± SD, *n* = 3 for each group. ^#^*P* < 0.05, ^##^*P* < 0.01 vs. sham; ^∗^*P* < 0.05, ^∗∗^*P* < 0.01 vs. MCAO.

To further identify whether JAK2/STAT6 signaling participated in IL-13-mediated M2 microglia polarization, the levels of JAK2, p-JAK2, STAT6, and p-STAT6 were measured by western blotting. As shown in Figure 7, higher levels of ratios of p-JAK2/JAK2 (*P* < 0.05, Figures 7(a) and 7(b)) and p-STAT6/STAT6 (*P* < 0.01, Figures 7(a) and 7(c)) were measured at 7 days in the SGD group compared to the MCAO group. These data implied that SGD led to an increase in IL-13 expression that induced polarized microglia, possibly through the JAK2/STAT6 pathway.

### 3.7. SGD Reduced Apoptotic Cell Numbers after CI/RP

**Figure 8:**
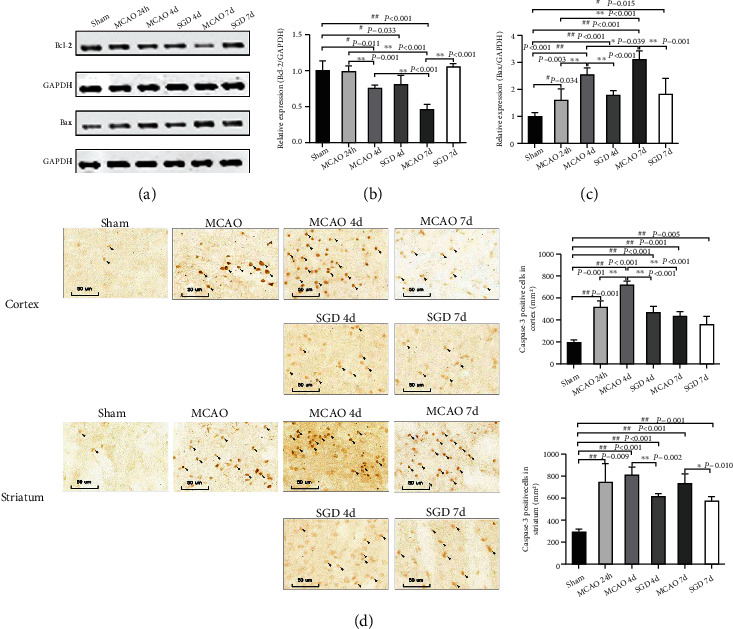
SGD reduced apoptotic cell numbers after CI/RP. We detected Bcl-2 and Bax levels by western blotting and apoptotic cells by immunohistochemistry (×200) of caspase-3 in the penumbra in the sham, MCAO, and SGD groups to assess the benefit of SGD after MCAO. (a) Representative bands of Bcl-2, Bax, and GAPDH. The statistical analysis of Bcl-2 (b) and Bax (c) was also presented graphically on the right side. *n* = 4 for each group. (d) Representative images of cell apoptosis (indicated by black arrows), and the statistical results of the number of positive cells were plotted as a histogram. Scale bar = 50 *μ*m, *n* = 3 for each group. ^#^*P* < 0.05, ^##^*P* < 0.01 vs. sham; ^∗^*P* < 0.05, ^∗∗^*P* < 0.01 vs. MCAO.

To develop the awareness of the effects of SGD on cell apoptosis after MCAO, western blotting of apoptosis-modulating protein Bcl-2 and Bax and immunohistochemistry of caspase-3 were used to label apoptotic cells [30] in the sham, MCAO, and SGD groups. As shown in Figure 8, a significant decline in the protein level of Bcl-2 at 4 d (*P* < 0.05) and 7 d (*P* < 0.01) after MCAO compared to the sham group was observed in this study (Figures 8(a) and 8(b)). At the same time, there is also an obvious increase in the expression of Bax at 24 h (*P* < 0.05), 4 d (*P* < 0.01), and 7 d (*P* < 0.01) post-surgery (Figures 8(a) and 8(c)). SGD treatment significantly elevated the level of Bcl-2 at 7 d (*P* < 0.01) and decreased the level of Bax at 4 d (*P* < 0.01) and 7 d (*P* < 0.01) post-surgery. Furthermore, the expression of caspase-3 in the cortex and striatum was elevated at 24 h, 4 h, and 7 h after treatment with MCAO compared to the sham group (*P* < 0.01, Figure 8(d)). There is statistical difference in caspase-3 levels at 4 d in the cortex (*P* < 0.01) and striatum (*P* < 0.01) and at 7 d in the striatum (*P* < 0.05) between the SGD and MCAO groups. In summary, SGD treatment inhibited apoptosis following CI/RP.

## 4. Discussion

In the present study, SGD-induced neuroprotection against cerebral ischemia-reperfusion injury and its underlying mechanisms were explored. The present data indicated that SGD was conducive to the alleviation of neurological deficits, an increase in M1-to-M2 microglia switching, and reduced cell apoptosis. From the concentrations of cytokines measured, we confirmed that SGD treatment significantly elevated IL-13 in MCAO rats, which was likely to activate the downstream JAK2-STAT6 signaling pathway to mediate the beneficial effects.

Our previous study demonstrated that SGD significantly increased IL-10 expression in the penumbra, reduced the activation of microglia and astrocytes, as well as the production of IL-1*β*, TNF-*α*, and monocyte chemotactic protein-1 (MCP-1) in the penumbra and serum, thus confirming the anti-inflammatory and neuroprotective feature of SGD after CI/RP [12]. However, the interaction in neuroprotection of SGD, activation of microglia/astrocytes, and pro- and anti-inflammatory cytokines expression lacked further investigation in our previous study. Recently, with related research developing in depth, it has been proven that activated microglia have pro- or anti-inflammatory effects, and this dual action is caused by the phenotypic change of microglia in a changing microenvironment in the injured brain [31–33]. Microglia of the M1-type release proinflammatory mediators that promote neuron apoptosis after CI/RP, while the M2 phenotype has an inhibitory effect on increased neuronal apoptosis via the production of neuroprotective factors [34–36]. Therefore, promoting the phenotypic shift of microglia from M1 to M2 is a crucial factor in aiding brain recovery after CI/RP.

To our excitement, we observed in this study that SGD administration not only reduced CD68^+^ M1 microglia and M1 markers (IL-1a, IL-1b, and IL-6) but also significantly elevated the levels of Arg-1^+^ M2 microglia and the M2 marker IL-10. The neurological scores and cell apoptosis were lower in the SGD group compared to the MCAO group. All these results indicated that the SGD intervention effectively induced microglia to polarize from M1 to the M2 phenotype, thus contributing to the improvement of the functional outcome. There is growing evidence that traditional Chinese medicine formulae can play a protective role against CI/RP injury by inhibiting the M1 phenotype and the M2 phenotype, and in fact, it is the active ingredients in traditional Chinese medicine that induce microglia polarization, such as ginsenoside [37, 38] of Modified Buwang powder. In this study, we first conducted HPLC analysis of SGD, which provided the quality control of SGD, namely, to reveal many compounds present in the chromatographic profile. These compounds have been considered as the most important bioactive constituents of SGD, and their content decided the efficacy and treatment effect of SGD [39], which could help explain the difference in the therapeutic effect of SGD treatment in different studies. According to the quantitative results of the HPLC analysis, the main components of SGD were ordered in importance as follows: paeoniflorin, glycyrrhizin, liquiritin, oxypaeoniflorin, isoliquiritin, albiflorin, and liquiritigenin. Luo et al. demonstrated that paeoniflorin transformed microglia from the M1 to M2 phenotype in the hippocampus, reduced the expression of proinflammatory mediators of M1, and increased the expression of anti-inflammatory cytokines of M2 to attenuate learning and memory damage in brain hypoperfusion rats [40]. Gao et al. found that glycyrrhizin possessed the characteristic of promoting M2 to M1 microglia polarization to facilitate recovery after traumatic brain injury [41]. Sun et al. drew the same conclusion in a neonatal hypoxic-ischemic rat model and confirmed this effect *in vitro* [42]. Liquiritigenin has also been reported to reduce cognitive impairment by regulating the microglia M1/M2 transition [43]. Therefore, SGD may contribute to brain repair after CI/RP through induction of M2 microglia.

As discussed above, induction of M2 microglia is associated with IL-4/IL-13 stimulation [44, 45]. Thus, we tested IL-4 and IL-13 expressions, and based on these results, we found that SGD significantly increased IL-13 content, possibly triggering an M1-to-M2 phenotype transition involved in this protective process. These findings are consistent with several studies that showed that an IL-13 supplement resulted in the phenotypic switching of microglia from M1 to M2 both *in vivo* and *in vitro* [46, 47]. Furthermore, what intrigues us is that IL-13, rather than IL-4, participated in this process. Previously, paeoniflorin was shown to regulate IL-13 expression alone to decrease inflammation in asthmatic mice [48]. Glycyrrhizin could influence IL-13 production in an LPS-induced macrophage cell line [49]. As a result, IL-13 is the activator in the M1 to M2 switch in rats treated with SGD after MCAO. This IL-13 elevation might be attributed to the enhanced proportion of T helper-2 (Th2) cells, the main source of IL-13. The Th2 response that secretes anti-inflammatory cytokines such as IL-4 and IL-13 was found to decrease after CI/RP [50], resulting in enhanced neuronal death [51, 52]. Th2 cells prove to ameliorate functional disturbance after CI/RP [53], and some bioactive components of SGD, such as paeoniflorin [54] and glycyrrhizin [55], have been reported to modulate Th2 response, thereby increasing the expression of IL-13. It is worth noting that the significant difference in the expression of IL-13 and M1/M2 microglia took place 7 days after the surgery. It is thus inferred that these beneficial changes caused by SGD might mediate a longer-term protective effect after CI/RP.

Previous studies have shown that IL-13 signals were through the IL-13 R*α*1 and activated JAK/STAT pathway, mediating inflammatory responses and cell survival in central nervous system disorders [56, 57]. A study suggested that IL-13 could activate the JAK1/STAT1 pathway to prevent neuronal death and restore brain functions after traumatic brain injury [58]. The STAT family is widely perceived as central switches for activating the polarization of M1/M2 [33], and STAT6 is perceived as the primary pathway involved in IL-13 response [59]. In our research, we examined the total and phosphorylation levels of JAK2 and STAT6 and found that the ratios of p-JAK2/JAK2 and p-STAT6/STAT6 rose remarkably in the SGD group. Hence, it was inferred that JAK2/STAT6 might be the downstream effector of IL-13 to change the M1 phenotype into the M2 phenotype, thus participating in the SGD-induced neuroprotective process.

There are some limitations of our study. First, HPLC analysis showed seven ingredients of SGD used in this study. In the future, we will compare the effects of SGD and Paeoniae Radix alba, Glycyrrhizae Radix et rhizoma, and monomeric components on neurological functions, anti-inflammatory factors, microglia polarization, and apoptosis *in vivo* and *in vitro*, thus identifying the most effective components in SGD that contribute to alleviation of CI/RP injury. Given that IL-13 could also activate other members of the Jak family, such as JAK1 [60], future studies tend to select IL-13 knockout rats as subjects or cover exogenous administration of IL-13 *in vitro*, in order to further confirm this research conclusion. Besides, IL-10 expression was also found enhanced after SGD treatment in this study. IL-10 could drive M2 microglia polarization as well [45], and it is currently unclear whether M2 microglia polarization is modulated by IL-4 alone or in concert with IL-10 after SGD treatment. This provides a new research direction for determining which becomes the promoter of M2 polarization in the protective mechanism of SGD.

## 5. Conclusions

In conclusion, the present findings revealed the favorable effects of SGD treatment in MCAO rats. In general, SGD significantly alleviated neurological disorders and suppressed cell apoptosis. Increased IL-13 expression may activate the JAK2/STAT6 signaling pathway to change microglia from the M1 to the M2 phenotype. As far as we know, this is the first study to investigate in detail the molecular mechanisms underlying the anti-inflammatory effect of SGD on CI/RP injury, that is, the IL-13-JAK2/STAT6 pathway eliciting M2 microglia promotion.

## Figures and Tables

**Table 1:** The forward primer sequences for qRT-PCR.

Gene	Sequence (5′-3′)
IL-4	GTACCGGGAACGGTATCCAC
IL-13	TATCGAGGAGCTGAGCAACATCA
IL-1a	GAGTCGGCAAAGAAATCAAGA
IL-1b	AATGCCTCGTGCTGTCTGA
IL-6	CACTTCACAAGTCGGAGGCT
IL-10	CCTGCTCTTACTGGCTGGAG

**Table 2:** The regression equations of seven analytes in SGD.

Analyte	Regression equation	*R* ^2^
Paeoniflorin	*y* = 0.0002*x* − 1.847	0.9931
Albiflorin	*y* = 0.00005*x* − 0.0341	0.9997
Oxypaeoniflorin	*y* = 0.0006*x* − 0.321	0.9993
Liquiritin	*y* = 0.0002*x* − 2.2489	0.997
Isoliquiritin	*y* = 0.00008*x* + 0.0536	0.9999
Glycyrrhizin	*y* = 0.0002*x* − 0.0712	0.9999
Liquiritigenin	*y* = 0.00002*x* − 0.0239	0.9997

**Table 3:** The contents of the seven analytes in SGD.

Analyte	Concentration (mg/mL)
Paeoniflorin	24.4386
Albiflorin	1.23946
Oxypaeoniflorin	3.957
Liquiritin	17.3426
Isoliquiritin	1.532432
Glycyrrhizin	18.47914
Liquiritigenin	0.09507

## Data Availability

All data of this study are available from the corresponding author Zhang Y if needed.
